# The odonate phenotypic database, a new open data resource for comparative studies of an old insect order

**DOI:** 10.1038/s41597-019-0318-9

**Published:** 2019-12-12

**Authors:** John T. Waller, Beatriz Willink, Maximilian Tschol, Erik I. Svensson

**Affiliations:** 1 Department of Biology, Lund University, SE-223 62 Lund, Sweden; 2 Present Address: Global Biodiversity Information Facility (GBIF), GBIF Secretariat Universitetsparken 15, DK-2100 Copenhagen Ø, Denmark; 3 Present Address: School of Biology, University of Costa Rica, San Jose, 11501-2060 Costa Rica; 4 Present Address: School of Biological Sciences, Zoology Building, University of Aberdeen, Tillydrone Avenue, Aberdeen, AB24 2TZ UK

**Keywords:** Phylogenetics, Biogeography, Evolutionary ecology, Behavioural ecology, Taxonomy

## Abstract

We present The Odonate Phenotypic Database (OPD): an online data resource of dragonfly and damselfly phenotypes (Insecta: Odonata). Odonata is a relatively small insect order that currently consists of about 6400 species belonging to 32 families. The database consists of multiple morphological, life-history and behavioral traits, and biogeographical information collected from literature sources. We see taxon-specific phenotypic databases from Odonata and other organismal groups as becoming an increasing valuable resource in comparative studies. Our database has phenotypic records for 1011 of all 6400 known odonate species. The database is accessible at http://www.odonatephenotypicdatabase.org/, and a static version with an information file about the variables in the database is archived at Dryad.

## Abstract (summary)

**Table:** 

Measurement(s)	Invertebrate Taxonomy • body length • wing • appendage morphology trait • mating behavior • flight behavior • habitat • ecozone • climate • sexual dimorphism • size • color • biological pigment
Technology Type(s)	digital curation
Factor Type(s)	phenotypes
Sample Characteristic - Organism	Zygoptera • Anisoptera
Sample Characteristic - Environment	stream • pond • wetland ecosystem • lake • river • ephemeral spring
Sample Characteristic - Location	South America • North America • Africa • Asia • Europe • Australia

Machine-accessible metadata file describing the reported data: 10.6084/m9.figshare.10321595

## Background & Summary

The Odonate Phenotypic Database is an online data resource for dragonfly and damselfly phenotypes (Insecta: Odonata). The database consists of a variety of morphological, life-history and behavioral traits, and biogeographical information collected from various sources in the literature. The database is not intended for species identification, but for comparative analysis of insect groups or to be combined with data from other taxonomic groups. The database is provided along with a large phylogenetic tree (1322 taxa, 21% of known odonates https://www.pugetsound.edu/academics/academic-resources/slater-museum/biodiversity-resources/dragonflies/world-odonata-list2/, accessed in November 2015): “The Odonate Super Tree”. This phylogenetic tree was constructed using DNA-sequences from GenBank in combination with a traditional (morphologically-based) odonate taxonomy as our backbone^1^.

Comparative analyses are becoming an increasing common part of evolutionary studies, as researchers attempt to bridge the gap between microevolutionary processes and macroevolutionary patterns^2–8^. Most comparative analyses require on high-quality phenotypic data collected from the literature, and often a large amount of time is spent collecting such data. Trait information can be obtained from measurements of live and field-caught individuals, museum specimens or literature sources but often important covariates are missing, such as behavioural information or habitat data. It is therefore in the interest of many of us working in the field to collect and curate such phenotypic data in a coherent fashion so that such data can be used in future studies and combined with multiple other sources of phenotypic information, particularly in light of the explosion in phylogenetic comparative methods the last decades^8,9^.

Paradoxically, as a community we have been much more successful at storing and cataloguing genotypes, and DNA-sequences often exist in GenBank for many species, but not even basic phenotypic data (such as body size) exist in an easily accessible form for many organismal groups. This lack of information is most likely due to there being no clear structure or well-established routines agreed upon in how to store phenotypic data and which aspects of phenotypes should be measured. Phenotypic databases, because of the high-dimensional nature of most phenotypes, are also very different from a genetic database such as GenBank. Recent calls for a new field of “phenomics” – i.e. obtaining high-throughput phenotypic data in a similar fashion as in genomics – will always have to prioritize what aspects of the phenotypes to measure^10,11^. There are many challenges in developing such a general and cross-taxonomic research programme in phenomics, in particular the difficulties of establishing general and operational phenotype ontologies between distantly related organisms across the Tree of Life^12,13^.

From an evolutionary viewpoint, phenotypes are arguably just as important and interesting as genotypes, if not more so^11,14,15^, as selection operates on phenotypes, regardless of their genetic basis^16^. Increasingly integrative research practices in evolutionary biology will need not only access to high-quality genomic, molecular and phylogenetic resources, but will also need high-quality phenotypic and biogeographic data, fossil information for time-calibration of phylogenetic trees and other general data provided by biodiversity informatics^17^. The difficulty of these tasks and the size of each individual project should ideally not prevent the establishment of accessible structures needed to store the data.

One way forward is to create taxon-specific phenotypic databases, as we have done here. Having such databases available that focus on a certain taxonomic group, also allows the recorded phenotypes to be tailored to fit the needs of the specific group and have the advantages that trait definitions are less ambiguous. Examples of such open databases with various forms of phenotypic, biogeographic and phylogenetic data include AmphiBIO for amphibian ecological traits^18^, panTHERIA^19^ and EltonTraits 1.0^20^ for birds and mammals, a global invasion atlas of birds^21^, Tree of Sex (a database on eukaryotic sex determination systems)^22^, a recent database on thermal developmental plasticity of reptiles^23^ and a global database on plants^24^. However, in the case of animals, such databases are largely focused on vertebrate groups, while the most speciose animal group – the insects – have few such open databases available. One exception is the Freshwater Biological Traits Database^25^ which contains trait data for North American macroinvertebrate taxa and includes habitat, life history, mobility, morphology, and ecological trait data, although not in all cases down to the species level.

**Fig. 1:**
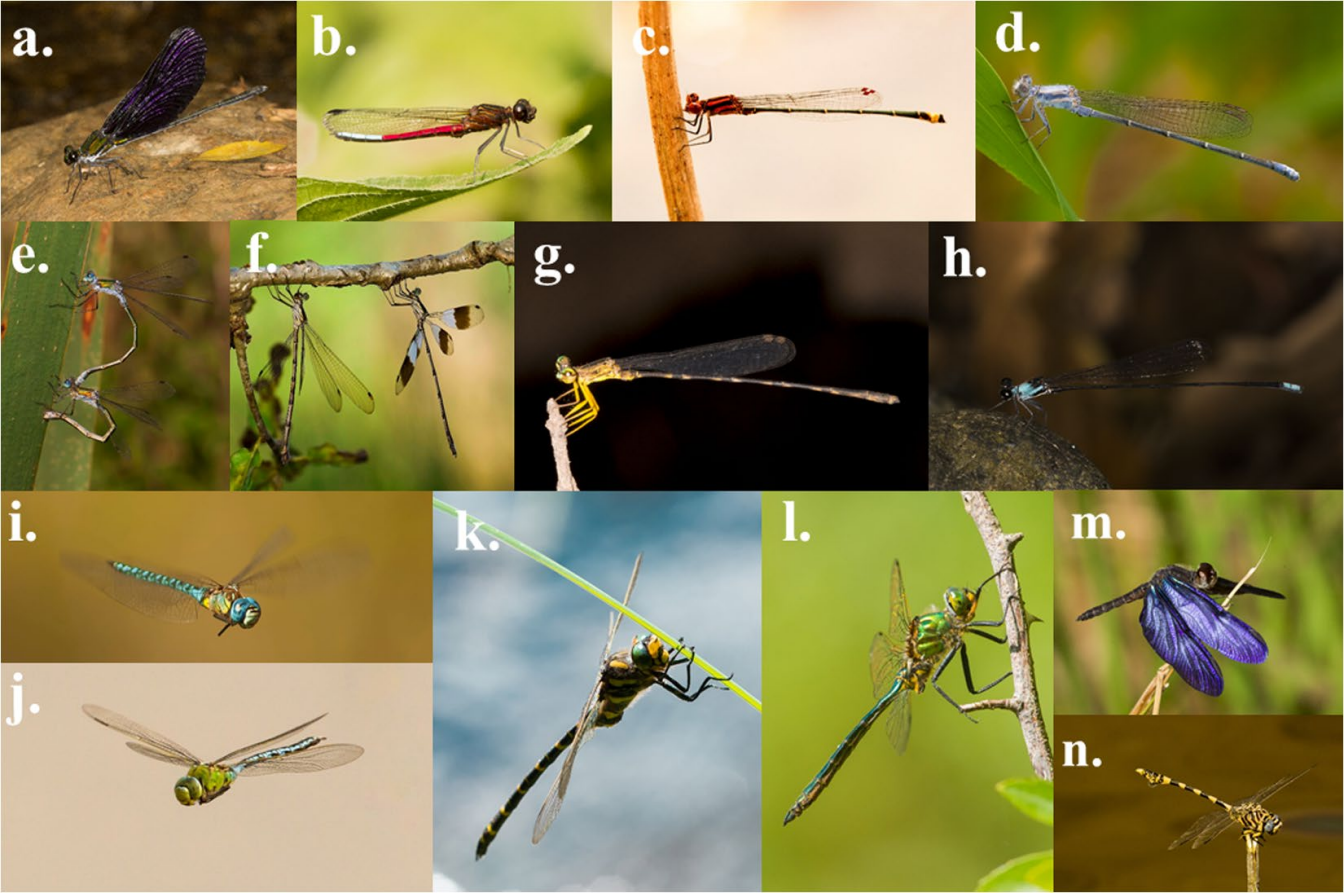
Phenotypic and taxonomic diversity of Odonata. Phenotypic and taxonomic diversity of 12 representative families of Odonata, an insect order which currently encompasses c. a. 6400 species and a total of 32 families. All 32 odonate families are included in our molecular and time-calibrated phylogeny (Fig. 2). (**a**) Family Calopterygidae: *Sapho orichalcea* (Cameroon, Africa, January 2017). (**b**) Family Chlorocyphidae: *Chlorocypha curta* (Cameroon, Africa, January 2017). (**c–d**) Family Coenagrionidae: (**c**) *Acanthagrion adustum* (Guyana, South America, January 2015). (**d**) *Argia moesta* (Texas, North America, April 2012). (**e**) Family Lestidae: *Lestes sponsa* (Sweden, Europe, July 2010). (**f**) Family Synlestidae: *Chlorolestes tessellatus* (Eastern Cape, South Africa, Africa, April 2010). (**g**) Family Platycnemidae: *Copera congolensis* (Cameroon, Africa, February 2017). (**h**) Family Protoneuridae: *Elattoneura balli* (Cameroon, Africa, January 2017). (**i–j**) Family Aeshnidae: (**i**) *Aeshna affinis* (Sweden, Europe, August 2010). (**j**) Anax imperator (Sweden, Europe, August 2015). (**k**) Family Cordulegasteridae: *Cordulegaster boltonii* (Sweden, Europe, July 2016). (**l**) Family Corduliidae: *Somatochlora metallica* (Sweden, Europe, June 2014). (**m**) Family Libellulidae: *Zenithoptera fasciata* (Guyana, South America, January 2015). (**n**) Family Gomphidae: *Ictinogomphus ferox* (Namibia, Africa, April 2017). All photographs by Erik Svensson.

We see taxon-specific phenotypic databases as becoming an increasingly valuable resource in comparative studies. Our research background and expertise is in the insect order Odonata (dragonflies and damselflies)(Fig. 1). To this end, we have collected data on 35 phenotypic, ecological and biogeographical variables that we see as useful to the research community (Online-only Table 1). Odonata is a relatively small insect order that currently consists of about 6400 species belonging to 32 families^1^. Odonata are morphologically highly conserved with respect to their overall external morphology (all species in this insect order have six legs and four wings), but they show considerable diversity in terms of wing and body colouration and shape (Fig. 1). Our database has at least some phenotypic records for 1011 of all 6400 known odonate species^1^ and a total of 3978 records (i.e., multiple records for many species, from different literature sources). The database is accessible at http://www.odonatephenotypicdatabase.org/.

## Methods

We have collected phenotypic data and data on habitats, climatic classifications and more coarse-grained biogeographic categories (e. g. ecozones^26^), from the scientific literature and from odonate field guides (Online-only Table 1). The field guides from which we obtained our phenotypic data are listed in Online-only Table 2. Phenotypes were scored by following a specific set of instructions for each variable that are described in an accompanying file that is uploaded alongside this paper as Supporting Material (“VariableDefinitions.pdf”) which is available on the Dryad Digital Repository^26^. The descriptions of each variable can be found in the Data Records section below and in Online-only Table 1. The construction of the Odonate Phylogenetic Super Tree has been described in detail elsewhere^1^.

Developing general and meaningful phenotype ontologies that are generally applicable across many taxa is a challenging task that is beyond the scope of this paper. The phenotypic variables (size, behaviours, wing and body colouration, colour patterns; Online-only Table 1) that we have assembled from the literature are not always easily and straightforward to translate to other, more well-studied insect groups, including the classical model organism *Drosophila melanogaster*, although our classifications are largely consistent with the recommendations given in the Ontobee database (http://www.ontobee.org/) and the Phenotype and Trait Ontology (PATO) database (http://www.ontobee.org/ontology/PATO)(Online-only Table 1). Clearly a lot of work is needed before we can develop generally applicable phenotypic ontologies across the entire class Insecta, let alone across more distantly related organismal groups^12,13^. All of our size measurements are given in millimetres (mm), although we acknowledge the fact that even a simple variable like wing length can be measured in different ways in different studies.

With respect to the ecological and geographic variables, our database is more easily connected and comparable with similar initiatives from other organismal groups. The ecozone variable for the distribution data of the different species follows the recent updates and classifications of Wallace’s classical zoogeographic regions by Holt *et al*.^27^. Our habitat classifications of water bodies (Online-only Table 1) are largely consistent with the environmental ontologies given in the Environment Ontology database (ENVO: http://www.ontobee.org/ontology/ENVO). We define *lakes*, *ponds*, *rivers* and *streams* in the same way as ENVO, whereas our classification of *Ephemeral* wetlands encompass both *ephemeral springs* (http://www.ontobee.org/ontology/ENVO?iri=http://purl.obolibrary.org/obo/ENVO_00000204) and *ephemeral rivers* (http://www.ontobee.org/ontology/ENVO?iri=http://purl.obolibrary.org/obo/ENVO_01000979).

## Data Records

The database has been deposited on the Dryad Digital Respository^26^, and additional material, including the code that is also uploaded on Github (see above) is also deposited with the most recent and updated database version at http://www.odonatephenotypicdatabase.org/. This data includes a PDF-file that describes each of the variables within the database and how they were collected (“VariableDefinitions.pdf”)^26^.

**Fig. 2:**
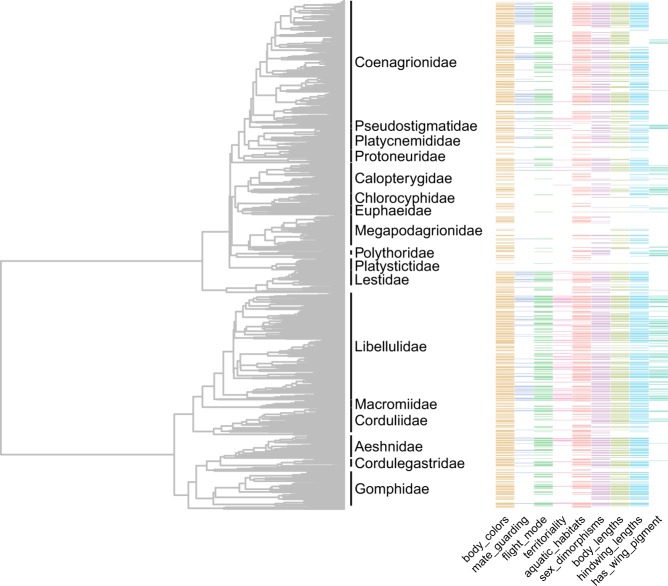
Our molecular time-calibrated phylogenetic tree, encompassing 1322 dragonfly and damselfly species. Some representative data is annotated on the tree showing coverage of some of the variables in the database. Absence of colour (=white) indicates absence of information for that particular taxon.

We note that our database contains information from only 1011 of all 6400 species, i. e. about 16% taxonomic coverage, and coverage varies among traits (Fig. 2). For instance, behavioural traits like male guarding of females and territoriality have considerably lower coverage than morphological traits like size and other phenotypic measurements (Online-only Table 1; Fig. 2). The reason for this is that morphological traits can easily be obtained also from dead specimens in museums, whereas behavioural traits would typically require time-consuming field studies of these insects^28,29^, particularly in the tropics where species diversity is high but where we still lack basic faunistic information even about which species occur in many countries^30^. It is particularly noteworthy that much data overall is lacking from several tropical damselfly families (Calopterygidae, Chlorocyphidae, Euphalidae, Megapodagrionidae, Polythoridae and Platystictidae) and some dragonfly families, particularly Gomphidae (Fig. 2). Our dataset illustrated in Fig. 2 should hopefully serve as a basis for targeted studies on these families.

Finally, we also note that information about the occurrence of female colour polymorphisms, which is an important feature of many odonate species and which have important roles in frequency-dependent sexual conflict^31–37^ is missing for many species and genera. Documenting the presence or absence of female colour polymorphism typically requires large sample sizes, particularly if one wishes to estimate the frequencies of certain morphs, such as male-mimicking females, and such information is typically available for mainly temperate species, such as those in the genera *Ischnura* and *Enallagma*^35,38^. There is clearly a need for more quantitative field studies and surveys to improve this situation and fill the missing gaps in our knowledge, particularly for tropical taxa.

## Technical Validation

All of the phenotypic data were collected from published field guides or reliable internet sources. The field guides are listed in Online-only Table 2. All the field guides have been published by respected odonatologists and experts on species identification. Our database is therefore not static, and additional data will be added as it becomes available. Researchers interested in contributing to this project are encouraged to contact the author for correspondence on how to incorporate new data. We will accept data both from already published sources (e. g. scientific papers) even if it has already been deposited in other databases such as Dryad, as well as data that is not intended to be published elsewhere, as long as it can be tailored to the format of the Odonate Phenotypic Database. Each species has a reference list, which lists the references from where the data were gathered, so it is possible to check each entry against these primary sources.

## Usage Notes

The database is intended to be used in future comparative analyses of odonate trait evolution. We therefore provide a previously published phylogenetic tree (Fig. 2) along with these phenotypic data. Previous questions that we have addressed in recent past using phylogenetic comparative methods and these and similar data include the relationship between latitude and wing pigmentation^39^ and the micro- and macroevolutionary dynamics of body size^1^. We have also investigated diversification dynamics (speciation and extinction rates) in relation to body size and wing pigmentation^1,39^. Body size is also a trait that is of interest for conservation biology, as extinction risk (as defined by IUCN redlist criteria) was recently demonstrated to be significantly related to body size in damselflies^40^. In addition, behavioural diversity (“ethodiversity”) is a neglected aspect of biodiversity and conservation biology^29^ and in the future, we hope to incorporate such information in this database.

Other interesting questions to pursue in even more depth in the future include the evolutionary and ecological dynamics of sexual conflict and its consequences for the maintenance of sex-limited colour polymorphism in females^31–36^, and how climatic niche conservatism^41,42^ can shape the evolution, ecology and biogeography of odonate range limits^43–46^.

## Data Availability

All code used to generate the website and Odonate Super Tree are available on Github (https://github.com/jhnwllr/shiny-server/tree/master/odonates).

## Online-only Tables

**Online-only Table 1:** Current species coverage (%) of the phenotypic and ecological variables as of the publication of this database. Superscripts at the different categories refer to relevant ontologies (when available) in the Ontobee database (http://www.ontobee.org/).

Category	Variables	Description	Coverage
Taxonomy^a^	GenusSpecies Genus Species Family SubOrder	Taxonomy of record. Names are taken from the World Odonata List	15%
Size (in mm)^b^	body_lengths forewing_lengths hind_wing_lengths	Size (in mm) of species record	13%
Body Colors^c^	body_colors body_colortypes body_patterns	Coloration and pattern information	15%
Behaviour^d^	mate_guarding flight_mode territoriality	Mating and flight behaviours	15%
Location and Habitat	continents aquatic_habitats climates ecozones^e^ habitat_openness	Biogeograpic, habitat and climate information from range maps	15%
Morphisms	sex_polymorphisms sex_dimorphisms geo_polymorphisms	Polymorphisms and sexual dimorphism	15%
Wing Pigment^f^ or Color^c^	has_wing_pigment wing_pigment_extent_discrete wing_pigment_extent_continuous wing_pigment_pattern wing_pigment_symmetry wing_pigment_dimorphism wing_pigment_color wing_pigment_placement wing_pigment_color_type female_has_wing_pigment female_wing_pigment_extent_discrete female_wing_pigment_extent_continuous female_wing_pigment_placement	Wing color and wing pigment variables	15%

Notes and links to relevant ontologies in Ontobee:

^a^Taxonomy: http://bioportal.bioontology.org/ontologies/EDAM?p=classes&conceptid=data_3028

^b^Size: http://www.ontobee.org/ontology/PATO?iri=http://purl.obolibrary.org/obo/PATO_0000117

^c^Colour: http://www.ontobee.org/ontology/PATO?iri=http://purl.obolibrary.org/obo/PATO_0000014

^d^Behaviour: http://semanticscience.org/resource/SIO_001195.rdf

^e^Ecozones: http://www.ontobee.org/ontology/ENVO?iri=http://purl.obolibrary.org/obo/ENVO_01000280

^f^Pigmentation: https://www.ebi.ac.uk/chebi/searchId.do?chebiId=CHEBI:26130.

**Online-only Table 2:** The primary references from which the data were collected. Most of these are field guides. Below is a list of resources that the database currently is based on.

Authors (year)	Title	Source
Theischinger and Hawking (2006)	*Complete Field Guide to Dragonflies of Australia*	^47^
Ozono and Futahashi (2012)	*Dragonflies of Japan*	^48^
Dijkstra and Lewington (2006)	*Field Guide to the Dragonflies of Britain and Europe*	^49^
Paulson (2009)	*Dragonflies and Damselflies of the West*	^50^
Samways (2008)	*Dragonflies and Damselflies of South Africa*	^51^
Tarboton and Tarboton (2002)	*A fieldguide to the Dragonflies of South Africa*	^52^
Heckman (2006)	*Encyclopedia of South American Aquatic Insects: Odonata-Anisoptera*	^53^
Paulson (2012)	*Dragonflies and Damselflies of the East*.	^54^
Bun *et al*. (2010)	*A Photographic Guide to the Dragonflies of Singapore*	^55^
Subramanian (2005)	*Dragonflies and Damselflies of Peninsular India: A Field Guide*	^56^
Hämäläinen and Pinratana (1999)	*Atlas of the Dragonflies of Thailand Distribution Maps by Provinces*	^57^
Herrara (2006)	*Dragonflies and damselflies of Middle America and the Caribbean*	^58^
Garrison *et al*. (2006)	*Dragonfly Genera of the New World: An Illustrated and Annotated Key to the Anisoptera*	^59^
Nair (2011)	*Dragonflies and Damselflies of Orissa and Eastern India*	^60^
Tarboton and Tarboton (2005)	*A fieldguide to the Damselflies of South Africa*	^61^
Sugimura *et al*. (2001)	*Dragonflies of the Japanese Archipelago in Color*	^62^
Lencioni (2006)	*Damselflies of Brazil: An illustrated identification guide*. *Volumes 1 and 2*.	^63^
Suhling and Martens (2007)	*Dragonflies and Damselflies of Namibia*	^64^
Askew (1988)	*The Dragonflies of Europe*	^65^
Dunkle (2000)	*Dragonflies through Binoculars: A Field Guide to Dragonflies of North America*	^66^
Manolis (2003)	*Dragonflies and Damselflies of California*	^67^
Abbott (2011)	*Damselflies of Texas: A Field Guide*	^68^
Dijkstra and Clausnitzer (2014)	*The Dragonflies and Dameselflies of Eastern Africa: Handbook for All Odonata from Sudan to Zimbabwe*	^69^
Tze-wai *et al*. (2011)	*The Dragonflies of Hong Kong*	^70^
Michalski (2015)	*The Dragonflies and Damselflies of Trinidad and Tobago*	^71^
Kompier (2015)	*A Guide to the Dragonflies and Damselflies of the Serra dos Orgaos South-eastern Brazil*	^72^
Marinov and Waqa-Sakiti (2013)	*An Illustrated Guide to Dragonflies of Viti Levu Fiji*	^73^
Polhemus and Asquith (1996)	*Hawaiian Damselflies: A Field Identification Guide*	^74^
Biggs (2000)	*Common Dragonflies of California*	^75^
Garrison *et al*. (2010)	*Damselfly Genera of the New World: An Illustrated and Annotated Key to the Zygoptera*	^76^
